# Cadmium is a stowaway on the zinc transporter AcZIP1 from *Agaricus crocodilinus*

**DOI:** 10.1007/s00203-025-04637-0

**Published:** 2026-01-06

**Authors:** Jan Sácký, Sára Melicharová, Irena Jochovičová, Vladimíra Svobodová Pavlíčková, Jan Borovička, Tereza Leonhardt

**Affiliations:** 1 Department of Biochemistry and Microbiology, University of Chemistry and Technology, Prague, Technická 3, Prague 6, 166 28 Czech Republic; 2 Institute of Geology of the Czech Academy of Sciences, Rozvojová 269, Prague 6, 16500 Czech Republic; 3 Nuclear Physics Institute of the Czech Academy of Sciences, Hlavní 130, Husinec- Řež, 25068 Czech Republic

**Keywords:** Agaricaceae, Mycelia, Toxicity, Heavy metals, Metal tolerance, Metal accumulation

## Abstract

Fungal activity significantly contributes to the turnover of minerals and other nutrients in the environment. Some fungi exhibit a remarkable ability to accumulate heavy metals; however, the molecular basis of this process remains poorly understood. This study investigates the metal tolerance and uptake capabilities of *Agaricus crocodilinus* mycelium, focusing on cadmium (Cd) and zinc (Zn). The mycelium was isolated from a naturally growing sporocarp that showed a Cd concentration of 149 mg kg⁻¹ dry weight, roughly 100-fold higher than typical mushroom levels. Mycelial metal accumulation assays demonstrated a linear relationship for Cd uptake (R^2^ = 0.9977) but a logarithmic relationship for Zn uptake (R^2^ = 0.9965), suggesting unregulated Cd uptake and regulated Zn uptake. Comparison of the *A. crocodilinus* Zinc-regulated transporter/Iron-regulated transporter-like Protein (ZIP) gene Ac*ZIP1*, showed homology with known high-affinity Zn transporters in *Agaricomycetes*. Functional expression of Ac*ZIP1* in *Saccharomyces cerevisiae* supported its role in Zn and Cd transport, with localization studies indicating its presence at the plasma membrane. Zn exposure reduced Ac*ZIP1* expression in mycelium ~ 3-fold, while Cd had no significant effect. This study improves our understanding of the possible means of metal uptake in *A. crocodilinus* and other *Agaricomycetes*.

**Supplementary Information:**

The online version contains supplementary material available at 10.1007/s00203-025-04637-0.

## Introduction

*Agaricus crocodilinus* Murrill (sect. *Arvenses*; syn. *A. urinascens* Singer) is a saprotrophic mushroom reported from temperate climates around the world that typically fruits in late summer–fall in grassland and woodland-edge habitats. It can be considered a cadmium (Cd) accumulator, reported to accumulate up to 249 mg Cd kg^− 1^ dry weight (Cocchi and Vescovi 1997), far exceeding typical levels found in most fungi (up to 5 mg kg^− 1^ dry weight) (Kalač 2019). This striking trait of Cd accumulation suggests the presence of an underlying Cd uptake and storage mechanisms in the fungus, as metal accumulation levels are often independent of background metal concentrations in the surrounding soil (Fletcher et al. 2024).

Cd and Zn are both group 12 metals that predominantly occur in the divalent state, with comparable ionic radii and coordination preferences. This biochemical resemblance enables Cd²⁺ to mimic Zn²⁺, interact with cysteine-rich motifs, and utilize cellular Zn transport systems and other metal homeostasis pathways (Brzóska and Moniuszko-Jakoniuk 2001; Robinson et al. 2021). In eukaryotes, Zn transport is primarily, but not exclusively, mediated by two families of proteins: the Cation Diffusion Facilitator (CDF) family, which exports metal ions from the cytoplasm to compartments (vacuole, endoplasmic reticulum) or extracellular space, and the Zinc-regulated transporter/Iron-regulated transporter-like Protein (ZIP) family, responsible for metal uptake (Kambe et al. 2015). Although prokaryotes generally possess only a few ZIP family genes in their genomes, eukaryotic genomes can contain up to nearly twenty ZIP family genes (Rogers et al. 2000).

Fungal ZIP family members are best studied in *Saccharomyces cerevisiae* (Desm.) Meyen (Eide 2006), which has four ZIP family proteins: high and low affinity ZRT1 and ZRT2, vacuolar ZRT3, and ZRT4 localized on the endoplasmic reticulum. Other ZIP transporters in fungi have been studied primarily in human fungal pathogens (Amich et al. 2014; Citiulo et al. 2012; Crawford and Wilson 2015) or plant pathogens (Martha-Paz et al. 2019), although some data are also available for *Agaricomycetes*: two high-affinity Zn uptake transporters, RaZIP1 from *Russula bresadolae* (Leonhardt et al. 2018) and SlZRT1 from *Suillus luteus* (Coninx et al. 2017), and low-affinity SlZRT2 from *S. luteus*, probably responsible for the redistribution of Zn from endoplasmic reticulum (Coninx et al. 2019).

Regarding Cd, direct reports of protein families capable of Cd uptake in fungi are limited (Robinson et al. 2021 and references therein), unlike in plants, where several protein families have been implicated in Cd transport besides the aforementioned ZIPs and CDFs: natural resistance-associated macrophage protein (NRAMP) family, Heavy Metal ATPase (HMA) family, Yellow Stripe-Like (YSL) protein family, ATP synthase (ATP)-binding cassette (ABC) transporter family and various Ca^2+^ channels (Liu et al. 2024; Xin et al. 2024 and references therein). In fungi, NRAMPs have been implicated in Cd uptake in *S. cerevisiae* (Liu et al. 1997) and other *Ascomycetes* (Diffels et al. 2006). Our knowledge of metal transport in *Basidiomycetes*, and particularly in the *Agaricomycetes*, remains limited, especially regarding the possible ways of Zn and Cd uptake that may contribute to metal accumulation traits exemplified by *A. crocodilinus*.

In this study, we investigated the contribution of the zinc transporter AcZIP1 to the Cd and Zn accumulation observed in *A. crocodilinus*. The transporter was identified by homologous cloning, and its role was further examined through functional characterization in a *S. cerevisiae* model and assessment of its expression in the native fungus. Together, these approaches provide a molecular perspective on how *A. crocodilinus* handles Cd and Zn, thereby contributing to our understanding of fungal metal homeostasis.

## Materials and methods

### Mycelia, yeast, and bacteria strains, vectors, and general growth procedures

Mycelial strain of *A. crocodilinus* ACRO2 was used in this study. This strain was isolated during our previous work, and details about its isolation source can be found within Sácký et al. (2025a). The mycelium was cultivated at 25 °C in the dark and maintained on potato dextrose broth (PD) agar plates (2 g potato extract, 10 g glucose, and 10 g agar per liter). Mycelial cultures in liquid PD medium (2 g potato extract and 10 g glucose per liter) were incubated at 25 °C without shaking in the dark.

The *S. cerevisiae* strains used in this study for the heterologous expression of Ac*ZIP1*, including the hemagglutinin-tagged (HA) version, were as follows: Cd-sensitive *ycf1*Δ strain DTY168 (Szczypka et al. 1994), Zn-sensitive *zrc1*Δ*cot1*Δ strain CM137 (MacDiarmid et al. 2000), and Zn-uptake deficient *zrt1*Δ*zrt2*Δ strain CM34 (MacDiarmid et al. 2000). Detailed information about the yeast genotypes is provided in Supplementary Data 2. The vector used for the constitutive expression of Ac*ZIP1* cDNA was the centromeric p416GPD (Mumberg et al. 1995). Yeasts were grown on synthetic defined (SD) agar plates at 30 °C. The SD agar plates contained 6.4 g L^–1^ of yeast nitrogen base without amino acids, 20 g L^–1^ d-glucose, 50 mg L^–1^ adenine hemisulfate, and 15 mg L^–1^ each of l-histidine, l-tryptophan, l-methionine and l-leucine, and 20 g L^− 1^ agar. Liquid yeast cultures were incubated at 30 °C and 150 rpm, unless otherwise indicated. *Escherichia coli* DH5α, used for plasmid amplification, was grown at 37 °C on Luria-Bertani agar supplemented with 150 µg mL^–1^ ampicillin.

### Mycelial metal tolerance assay

The metal tolerance assay of the *A. crocodilinus* mycelium ACRO2 was performed as previously (Sácký et al. 2019); however, mycelial cultures were grown in liquid PD medium in polypropylene 90-mm Petri dishes, instead of solid agar plate cultures. Briefly, a ~ 3 mm³ agar block of a 30-day-old mycelia was floated on 20 mL of liquid PD medium in a Petri dish. The medium contained various concentrations of Cd^2+^ (1, 2, 3, 4, 5, 7.5, 10 µM as CdCl_2_), Zn^2+^ (50, 100, 150, 200, 300 µM as ZnCl_2_), or no metal addition. The mycelial cultures were incubated for 21 d in the dark without shaking. Mycelium was harvested, frozen at -80 °C, lyophilized, and weighed. Residual growth (%) was calculated using the formula: (*m*_t_/*m*_c_) × 100%, where *m*_t_ is metal-treated mycelium, and *m*_c_ is control mycelium. The experiment was performed on the single mycelial isolate in biological triplicates, and the resulting residual growth values were averaged.

### Mycelial metal uptake assay

The metal uptake assay of the *A. crocodilinus* mycelium ACRO2 was performed as previously (Sácký et al. 2019). Briefly, a ~ 3 mm³ agar block of 30-day-old mycelia was floated on 20 mL of liquid PD medium in a Petri dish with no metal addition, and the mycelial cultures were grown for 21 d in the dark without shaking. Following the 21-day incubation period, the mycelium was transferred to a new Petri dish containing 20 mL of fresh liquid PD medium, supplemented with the appropriate metals (50, 100, 250 µM Zn^2+^ as ZnCl_2_ or 0.5, 1, 2.5, 5 Cd^2+^ as CdCl_2_) and cultured for an additional 72 h. To minimize interference from metals adsorbed on the mycelial cell walls, the mycelium was treated with 5mM ethylenediaminetetraacetic acid (EDTA) as previously described (Sácký et al. 2019). The washed mycelium was then lyophilized, weighed, and digested with 0.5 mL of 65% HNO_3_ for 24 h. The sample volume was adjusted to 10 mL with distilled water, and atomic absorption spectrometry (AAS; Central Laboratories, UCT Prague) was used to measure the metal content. Metal uptake experiments were conducted on a single mycelial isolate with three biological replicates, and the metal uptake values were averaged.

### Identification, PCR amplification, and cloning of Ac*ZIP1* cDNA and its HA-tagged variant

To design primers for the heterologous cloning of the Ac*ZIP1*, the following steps were used: first, BLASTp searches were performed on the UniProtKB (The UniProt Consortium 2025) with the target database set to UniProtKB reference proteomes + Swiss-Prot, restricted to *A. bisporus* var. *burnettii*, with previously characterized ZIPs from *Agaricomycetes* as query; then, the returned putative protein sequence AbZIP1 (UNIPROT: K5XEQ4) was used to retrieve the corresponding genomic sequence (GenBank: XM_007327467.1) from the *A. bisporus* var. *burnettii* JB137-S8 genome (Morin et al. 2013) at GenBank, NCBI (GenBank: AEOL00000000.1).

*A. crocodilinus* genomic DNA was isolated using the NucleoSpin Plant II Kit (Macherey-Nagel) and 20 mg of freeze-dried sporocarp tissue from collection B1081a/PRM 952,371 (Sácký et al. 2025a). Complete genomic DNA sequences of Ac*ZIP1* and adjacent regions were obtained using the GenomeWalker Universal Kit (Clontech) and primers, based on the *A. bisporus* Ab*ZIP1* sequence, designed as described above (primer sequences can be found in Supplementary Data 1). The resulting DNAs were sequenced (Eurofins) and used to design primers to amplify Ac*ZIP1* cDNA. To this end, first, total RNA was isolated from 20 mg of freeze-dried sporocarp tissue from collection B1081a/PRM 952,371 (Sácký et al. 2025a) by using RNeasy Plant Mini Kit (Qiagen) and converted to cDNA using the High-Capacity cDNA Reverse Transcription Kit (Applied Biosystems) with 0.5 µg of the total RNA. All PCR reactions were performed with Q5 polymerase (New England Biolabs). In silico translation of the cDNA was confirmed by reciprocal BLASTp search against UniProtKB. The annotated Ac*ZIP1* gene sequence was deposited in GenBank (GenBank: PQ160893.2).

The HA: Ac*ZIP1* construct was produced by PCR amplification from Ac*ZIP1* cDNA using primers Ac*ZIP1*-HATAG-F and Ac*ZIP1*-p416-InR (Supplementary Data 1). Both the Ac*ZIP1* and *HA*:Ac*ZIP1* PCR products were subsequently cloned into BamHI-linearized p416GPD vector with the In-Fusion HD Cloning Kit (Takara). Plasmids were transformed into yeasts by the LiAc/SS carrier DNA/PEG method of Gietz et al. (2007).

Unless specified, all commercial kits were applied following the protocols provided by the manufacturers.

### Functional complementation and accumulation assays in *S. cerevisiae*

Both assays were performed as described in Sácký et al. (2025a). Briefly, for metal tolerance assays, the mid-log cultures of *S. cerevisiae* transformants were adjusted to an optical density at 590 nm (OD_590_) of 0.2. Then, 4 µL of serial decimal dilutions of the cell suspension were spotted on SD agar plates supplemented with various concentrations of Cd^2+^ (1, 2.5, 10 µM as CdCl_2_) or Zn^2+^ (10, 100, 250 µM as ZnCl_2_), or without metal addition. The plates were incubated at 30 °C for 3 days and then photographed.

For the metal accumulation assays in SD medium, *S. cerevisiae* transformants were pre-grown in 40 mL SD medium to an OD_590_ of 1.6–1.8, then Cd^2+^ (1, 2.5, 10 µM as CdCl_2_), or Zn^2+^ (10, 100, 250 µM as ZnCl_2_), or no metal was added and the cultures were further incubated for 1 h at 30 °C, 150 rpm. To ensure that *zrt1*Δ*zrt2*Δ cells harboring the empty plasmid p416GPD were as viable as Ac*ZIP1*-transformed cells, SD medium was supplemented with 10 µM Zn^2+^. After incubation with or without metals, the protocol from Sácký et al. (2025a) was followed, i.e. cells were separated from the medium by centrifugation (3,000 × g, 2 min, 25 °C), washed twice with 10 mL of 5mM ethylenediaminetetraacetic acid (EDTA), digested by nitric acid and metal contents of the digestate was analyzed by AAS (Central Laboratories, UCT Prague).

### Immunofluorescence microscopy

For the preparation of the microscopy samples, the general protocol by Leonhardt et al. (2018) was followed. Yeast strain *zrt1*Δ*zrt2*Δ transformed with p416GPD containing the *HA*:Ac*ZIP1* construct or empty p416GPD were inoculated into 10 mL of SD medium with 10 µM Zn^2+^ to ensure even growth and incubated at 28 °C, 120 rpm. After reaching OD_590_ of 0.5, cells were fixed with 4.5% formaldehyde (Thermo Scientific, USA) for 20–30 min at room temperature with gentle shaking. Fixed cells were centrifuged (3 min, 3,000 × g) and washed with buffer A (1.2 M sorbitol, 0.1 M potassium phosphate, 1% 2-mercaptoethanol, pH 6.5). The cell pellet was resuspended in 0.5 mL buffer A with 10 mg mL^− 1^ driselase (Sigma Aldrich), and the mixture was incubated for 30 min at room temperature to allow digestion of the cell wall. Digestion was observed by light microscopy. The cells were centrifuged (3 min, 3,000 × g) and washed twice with buffer B (1.2 M sorbitol, 0.1 M potassium phosphate, pH 6.5). The resulting spheroplasts were incubated with fluorescein isothiocyanate (FITC)-labeled mouse monoclonal antibody anti-HA-FITC (1 mg mL^− 1^, Sigma-Aldrich) at a 1:300 ratio (v/v) in phosphate-buffered saline (PBS) supplemented with 0.2% bovine serum albumin for 16 h at 4 °C. In parallel, spheroplasts were also prepared without antibody staining. The spheroplasts were centrifuged (3 min, 3,000 × g) and washed twice with PBS. Finally, the spheroplasts were stained with 1 µg mL^− 1^ 4′,6-diamidino-2-phenylindole (DAPI) (Sigma Aldrich, USA) in the dark (10 min, 25 °C) and washed once with PBS. Samples were mounted on glass slides and immediately observed using an Olympus IX83 fluorescence microscope with a 100× objective and a Hamamatsu Orca Flash 4.0 V3 camera. Images were processed using CellSens software (Olympus).

### Relative quantification of Ac*ZIP1* in mycelial isolate

To assess Ac*ZIP1* metal responsiveness, reverse transcription quantitative PCR (qRT-PCR) was used to examine the mRNA levels in metal-exposed mycelia, following the procedures in Sácký et al. (2025a). Mycelial strain *A. crocodilinus* ACRO2 was first pre-grown from a ~ 3 mm³ agar block in 20 mL of liquid PD medium in a Petri dish for 21 d. Next, the medium was exchanged for 20 mL of fresh liquid PD medium, and the mycelium was incubated for 24 h. For the metal exposition, the culture medium was supplemented with various concentrations of Zn^2+^ (100, 250 µM as ZnCl_2_) or Cd^2+^ (1, 2.5 µM as CdCl_2_) and further incubated for 24 h. Mycelium grown in the same way but without additional metals served as a control.

Mycelia were frozen at -80 °C, lyophilized, ground with a mortar and pestle, and immediately used for total RNA isolation using a RNeasy Plant Mini Kit (Qiagen), with 20 mg of the material per isolation reaction. To remove residual DNA from the RNA samples, RNase-Free DNase Set (Qiagen) was used. Finally, cDNA was prepared using the High-Capacity cDNA Reverse Transcription Kit (Applied Biosystems) from 0.25 µg of the total DNA-free RNA.

Quantitative RT-PCR was performed using gene-specific primers (Supplementary Data 1) as described previously (Sácký et al. 2025a). The β-tubulin gene (Ac*TUB2*, GenBank: PV055698.1) served as the reference. Amplification efficiencies (*E*) were 114% for Ac*ZIP1* and 89% for Ac*TUB2*. All experiments were conducted in three biological replicates, each analyzed in technical duplicate. The mean *Cq* values of technical duplicates (reported in Supplementary Data 3) were used to calculate expression ratios between control samples (no metal exposure) and metal-treated samples according to the 2^^−ΔΔCt^ method (Pfaffl 2001). Ratios were log₂-transformed to represent fold changes in gene expression.

### Statistical methods and analyses

**Fig. 1:**
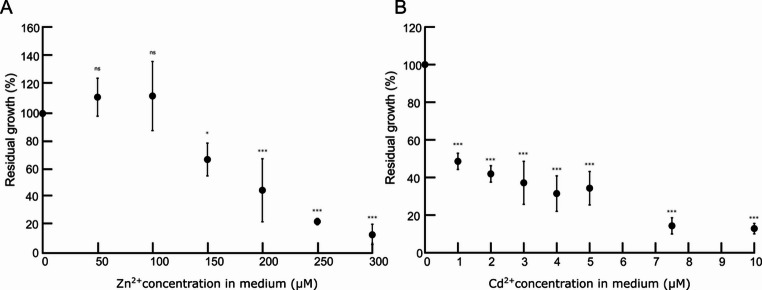
Zn (**A**) and Cd (**B**) tolerance assays of *A. crocodilinus* mycelium. Mycelia were exposed to the indicated metal concentrations for 21 d. Metal tolerance, expressed as residual growth (% of control dry weight), decreased with increasing metal concentration, showing higher sensitivity to Cd than to Zn. Each data point represents the mean of three biological replicates ± SD. ns – no significant inhibition compared to control; * - significant inhibition compared to control (*p* < 0.05), *** - highly significant inhibition compared to control (*p* < 0.001) as determined by one-way ANOVA with Tukey’s HSD post-hoc test

**Fig. 2:**
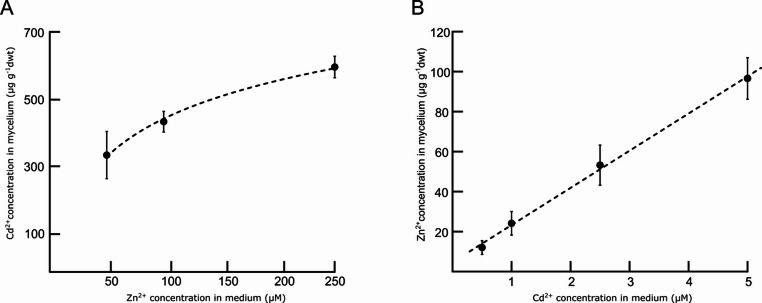
Zn (**A**) and Cd (**B**) uptake assay of *A. crocodilinus* mycelium. 21-day-old mycelia were exposed to sub-lethal concentrations of the metals for 3 d. For Zn, the metal uptake was slowing down at the higher Zn concentrations. For Cd, the uptake was still linear even at a relatively high Cd concentration. Each data point represents the mean values of three biological replicates ± SD. The dashed lines indicate the respective statistical models, logarithmic for Zn (R^2^ = 0.9965), linear for Cd (R^2^ = 0.9977)

In the mycelial metal tolerance experiments (Fig. 1), measured biomass values were normalized to the control (no metal added) set to 100%. The normalized values were then averaged, and standard deviation (SD) was calculated for each data point. One-way ANOVA (*p* < 0.05), followed by Tukey’s post-hoc test with homogeneous groups, was performed in Statistica 14.0 to assess the difference of the treated vs. control group. In the mycelial metal accumulation experiments (Fig. 2), measured values were averaged, and SD was calculated for each data point. The data were then fitted with linear or logarithmic models in Microsoft Excel, and the goodness of fit was evaluated comparing the resulting R² values. In the yeast metal accumulation experiments (Fig. 5), measured values were averaged, and SD was calculated for each data point. One-way ANOVA (*p* < 0.05), followed by Tukey’s post-hoc test with homogeneous groups, was performed in Statistica 14.0. In the gene expression experiment (Fig. 7), the log_2_ values were averaged and SD was calculated for each data point. One-way ANOVA (*p* < 0.05), followed by Tukey’s post-hoc test with homogeneous groups, was performed in Statistica 14.0.

## Results

### Metal tolerance and metal uptake assays in the mycelium isolate of *A. crocodilinus*

The growth of *A. crocodilinus* mycelium ACRO2 was adversely affected by increasing concentrations of both Zn and Cd (Fig. 1; detailed data are provided in Supplementary Data 3). Under control conditions (no metal added), the average dry biomass across all experiments reached 28.5 mg. Exposure to 200 µM Zn reduced biomass production to approximately 10 mg, while 1 µM Cd resulted in an average biomass of 17 mg. Thus, growth was reduced by nearly 50% at 200 µM Zn and 1 µM Cd. At higher concentrations, at 300 µM Zn and 10 µM Cd, the mycelium was barely viable, with average biomass yields of 3 mg and 5 mg, respectively, which is below 15% of control for both metals. These results indicate a pronounced sensitivity of the isolate to Cd compared to other fungal species, which is notable given the substantial Cd accumulation previously observed in the sporocarps. This apparent discrepancy suggests that Cd accumulation in *A. crocodilinus* may not stem from an inherently Cd-tolerant trait. The metal-tolerance assay also identified 250 µM Zn and 5 µM Cd as sublethal concentrations for subsequent experiments, as the mycelium was still growing above 20% of control. Although mycelial growth showed a slight, non-significant increase at 50 µM and 100 µM Zn, suggesting a potential hormesis effect, the differences were not statistically significant (one-way ANOVA followed by Tukey’s HSD test).

The accumulation of metals from the mycelium isolate was assessed under laboratory conditions by measuring the total metal content in the grown mycelia exposed to different metal concentrations for 3 d. As can be seen from Fig. 2, Zn uptake was well correlated with a logarithmic model (R^2^ = 0.9965), indicating slowing down of the metal uptake with increasing metal concentration in the medium. Contrary, on higher, but still sub-lethal Cd concentrations, the Cd uptake was still linear (R^2^ = 0.9977), suggesting no apparent regulation of the Cd uptake.

### In Silico characterization of *A. crocodilinus* AcZIP1

**Fig. 3:**
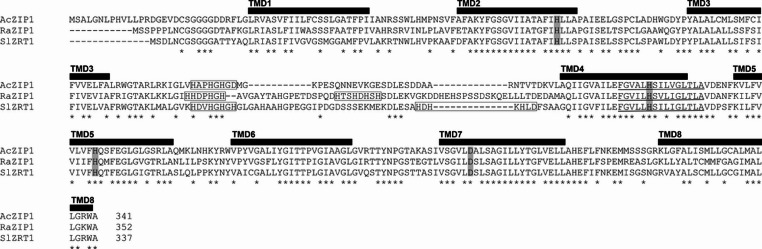
Amino acid sequence comparison of functionally characterized ZIP transporters from *Agaricomycetes*. Alignment of the protein sequences of AcZIP1 (GenBank: XED94869.2), RaZIP1 (GenBank: AMD82963.1), and SlZRT1 (GenBank: KIK44668.1). Black boxes indicate the positions of the predicted transmembrane domains (TMDs). The amino acid residues in the putative Zn-binding site H85, H197, H221, and D286 are marked by grey shading. ZIP signature sequence in TMD4 is underlined. The putative metal-sensing domains between TMD3 and TMD4 are boxed. The asterisks mark 100% identity in the AA sequence

To isolate the Ac*ZIP1* gene and transcript, knowledge of its close but uncharacterized ortholog from *A. bisporus* was used to design primers, allowing for the isolation of Ac*ZIP1* by homology cloning and GenomeWalking library. Once the protein sequence was established, it was compared with other ZIP proteins from *Agaricaceae*. The putative 341-AA AcZIP1 showed more than 50% identity with the two characterized *Agaricomycetes* high-affinity zinc transporters RaZIP1 (GenBank: AMD82963.1) and SlZRT1 (GenBank: KIK44668.1) as can be seen from the alignment in Fig. 3. AcZIP1 shares structural motifs typical of ZIP proteins: a very short C-terminal tail, eight conserved transmembrane domains (TMDs), and a histidyl-rich cytoplasmic loop of variable size between TMD3 and TMD4, and with considerable sequence variation as can be seen from the lack of asterisks in Fig. 3. Histidyl residues implied in Zn sensing in the loop are also present, however, AcZIP1 has a much shorter loop between TMD3 and TMD4 and only a single putative metal sensing domain (HxxHxHxD; x for any AA) compared to two domains in RaZIP1 (HxDxHxH, HxxHxHxH) and SlZRT1 (HxxHxHxH, HxHxHxD). TMD4 also contains the ZIP signature sequence, underlined in Fig. 3. The presence of conserved-in-ZIPs histidyls in the predicted TMD4 (H197) and TMD5 (H221) was also identified in AcZIP1. Overall, the analysis places AcZIP1 firmly into the ZIP protein family.

### Functional expression of Ac*ZIP1* in *S. cerevisiae*

**Fig. 4:**
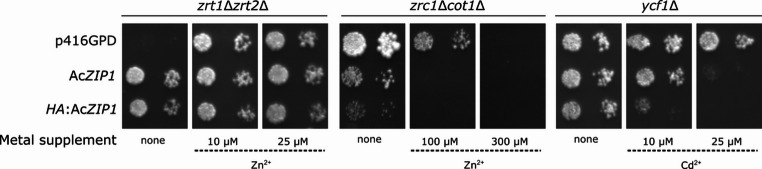
Growth of *S. cerevisiae* strains expressing Ac*ZIP1* and its HA-tagged variant. Phenotypes of the yeast strains are designated at the top of each panel, and the transformed construct is indicated on the left. Cultures were serially diluted ten times and spotted onto SD agar plates with or without metal supplement (indicated at the bottom of the panels). Images were taken after a 3-day incubation. Metal-uptake-deficient cells (*zrt1*Δ*zrt2*Δ) transformed with Ac*ZIP1*, show normal growth at plain SD medium (contains ~ 2.5 µM Zn^2+^), metal-sensitive cells (*zrc1*Δ*cot1*Δ and *ycf1*Δ) transformed with Ac*ZIP1*, show increased sensitivity compared to empty plasmid-only transformants

*S. cerevisiae* strains *zrt1*Δ*zrt2*Δ, *zrc1*Δ*cot1*Δ, and *ycf1*Δ were transformed with Ac*ZIP1*, its HA-tagged variant, or with empty p416GPD, which served as a control. Under our cultivation conditions, the *zrt1*Δ*zrt2*Δ cells transformed with empty p416GPD required an additional 10 µM Zn^2+^, and, as shown in Fig. 4, Ac*ZIP1* was able to fully complement the Zn-uptake deficiency phenotype without any Zn addition (standard SD medium contains 2.5 µM Zn^2+^). Ac*ZIP1* expression in the Zn-sensitive *zrc1*Δ*cot1*Δ strain resulted in inhibited growth on SD medium plates supplemented with Zn (Fig. 4). In addition, decreased growth of Ac*ZIP1*-transformed cells was also observed on SD medium plates without any Zn supplementation. Compared to the yeast that carried the empty plasmid p416GPD, Ac*ZIP1* expression impaired the growth of the strain of *ycf1*Δ sensitive to Cd already in the presence of 25 µM Cd^2+^ (Fig. 4). The tagging of Ac*ZIP1* with HA increased the sensitivity of *zrc1*Δ*cot1*Δ cells to Zn and *ycf1*Δ cells to Cd. These observations, i.e., complementing Zn-uptake deficiency and conferring increased Zn/Cd sensitivity, were congruent with the supposed function of AcZIP1 as an uptake transporter.

### Metal accumulation in *S. cerevisiae* strains expressing Ac*ZIP1*

**Fig. 5:**
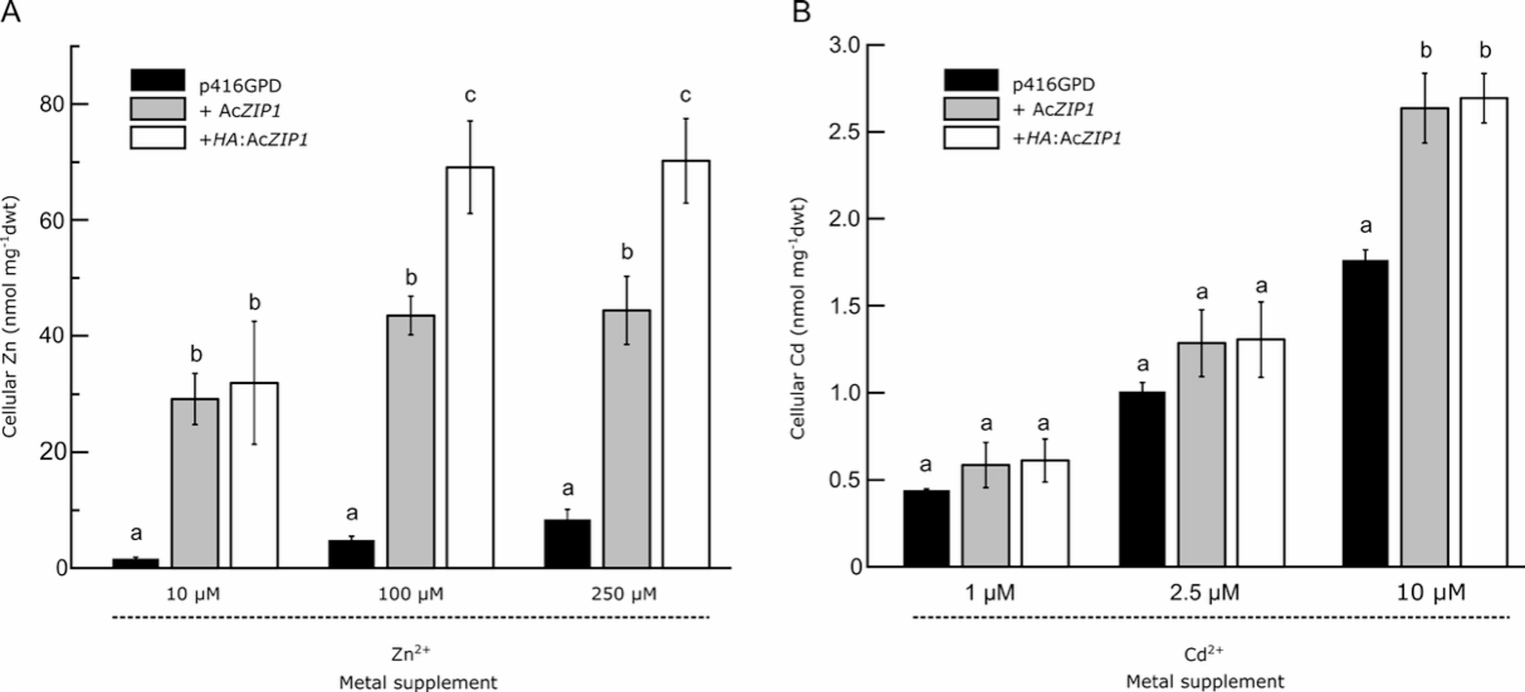
Metal accumulation in Ac*ZIP1-*expressing *S. cerevisiae*. Zn accumulation (**A**) was assessed in *zrt1*Δ*zrt2*Δ cells transformed with Ac*ZIP1* or *HA*:Ac*ZIP1*. Cd accumulation (**B**) was assessed in *ycf1*Δ yeast transformed with Ac*ZIP1* or *HA*:Ac*ZIP1*. Yeast cells transformed with Ac*ZIP1* or *HA*:Ac*ZIP1* were incubated for 1 h in the presence of the indicated metal concentrations. p416GPD-transformed cells were used as control. Cellular Zn and Cd was measured by AAS and the plotted values represent the average of at least three biological replicates ± SD. Letters above bars represent significant differences (*p* < 0.05, ANOVA followed by Tukey’s HSD test)

As shown in Fig. 5, Ac*ZIP1*-transformed *S. cerevisiae zrt1*Δ*zrt2*Δ cells accumulated an order of magnitude more Zn in the presence of 10 µM, 100 µM, and 250 µM Zn^2+^ compared to control cells transformed with p416GPD. Similarly to the experiment performed on solid media, the HA-tagged Ac*ZIP1* enabled the cells to accumulate even more Zn (but not Cd). There was no increase in Zn accumulation in Ac*ZIP1-*transformed cells from 100 µM to 250 µM Zn^2+^. Regarding Cd, expression of Ac*ZIP1* in *S. cerevisiae ycf1*Δ cells just about doubled Cd uptake only when 10 µM Cd^2+^ was added to the media and not at lower concentrations. A 10-fold difference was observed with p416-only transformed yeast cells and Ac*ZIP1*-expressing cells, suggesting a substantial contribution of Ac*ZIP1* towards Zn accumulation in the yeast model, unlike for Cd, where the difference in accumulation was not even statistically significant for both 1 µM and 2.5 µM. The not-even-one-fold difference at 10 µM Cd was statistically significant but suggested that there must be a much higher Cd concentration in the medium to “push through” the AcZIP1 transporter.

### Localization of AcZIP1 in *S. cerevisiae*

**Fig. 6:**
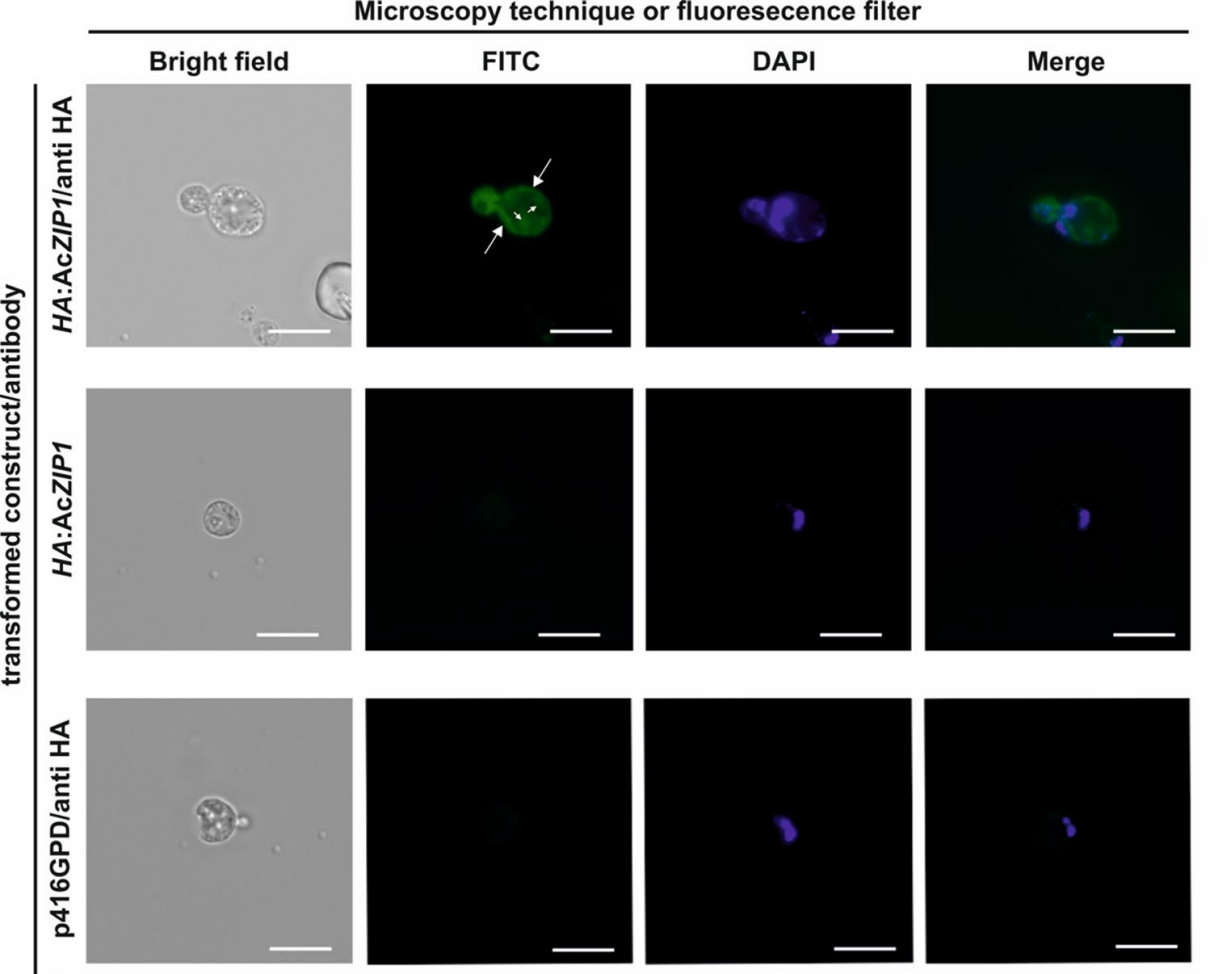
Fluorescent microscopy images of *HA*:Ac*ZIP1* expressed in *S. cerevisiae zrt1*Δ*zrt2*Δ dual labeled with anti-HA FITC (green fluorescence) and DAPI (blue fluorescence). Heterologously expressed Ac*ZIP1* localizes to the cytoplasmic membrane. The first row represents *HA*:Ac*ZIP1-*expressing cells with antibody staining, the second row shows images of *HA*:Ac*ZIP1-*expressing cells without antibody staining, and the third row represents control *zrt1*Δ*zrt2*Δ cells without *HA*:Ac*ZIP1* expression with antibody staining. Large arrows point to the cytoplasmic membrane located on the cell periphery. Small arrows point to the cytoplasmic membrane invaginations. The scale bars represent 10 μm

Immunofluorescent microscopy of the *S. cerevisiae zrt1*Δ*zrt2*Δ protoplasts expressing *HA*:Ac*ZIP1* was used to confirm the presumed localization on the plasmatic membrane. The anti-HA-FITC staining detected a FITC fluorescence signal at the protoplast periphery, confirming the localization of the heterologously expressed Ac*ZIP1* at the plasma membrane; however, certain irregularities and invaginations of the plasmatic membrane can be seen (Fig. 6). This pattern was consistent across the majority of the observed protoplasts.

### Analysis of Ac*ZIP1* gene expression in the mycelium of *A. crocodilinus*

**Fig. 7:**
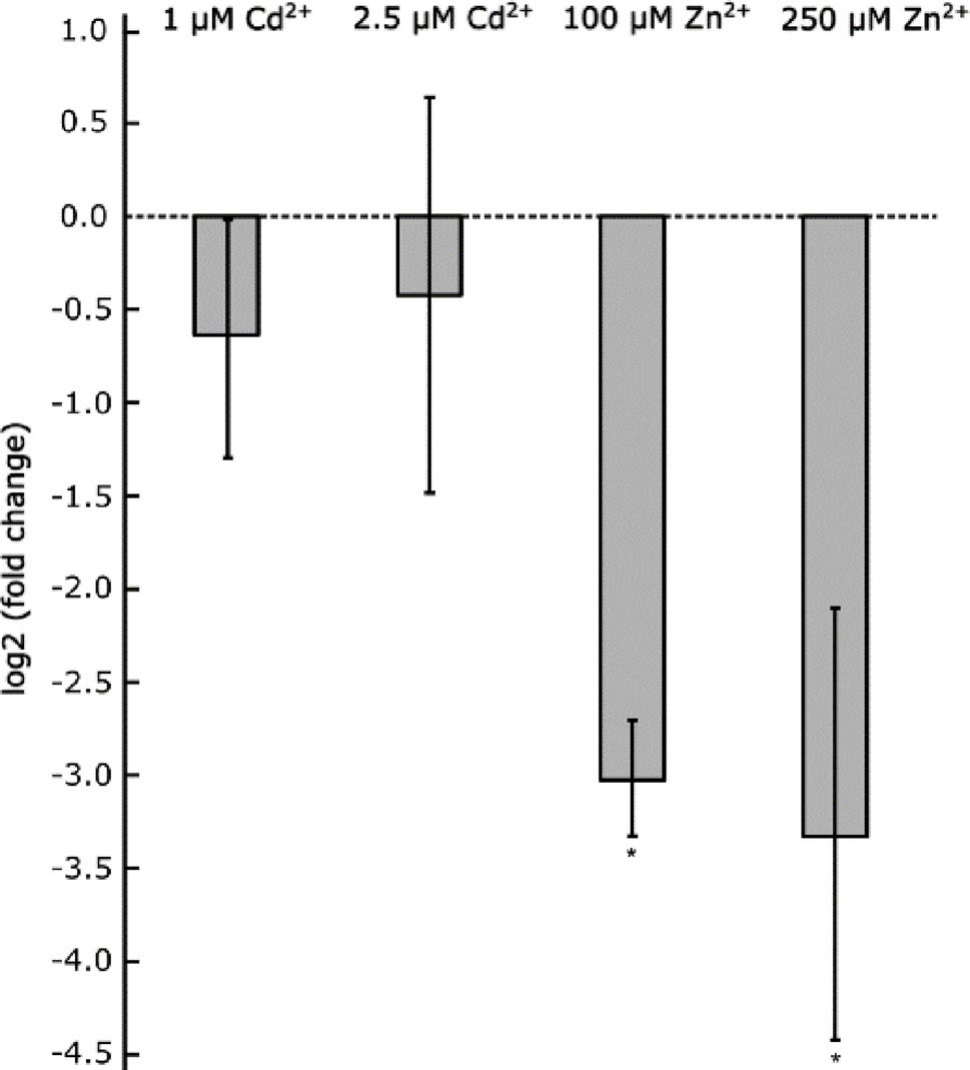
Ac*ZIP1* expression decreases under Zn exposure. Mycelium was precultivated in liquid PD medium for 21 d and then subjected to metals at the indicated concentrations for 24 h. Gene expression was measured by qRT-PCR and expressed as fold change of mRNA levels of Ac*ZIP1* (metal-exposed relative to non-exposed mycelia) with Ac*TUB2* as a control gene. The dashed line denotes the relative expression of Ac*ZIP1* when no metal was added. Values are plotted as averages of technical duplicates of three biological replicates ± SD. Asterisks below bars indicate statistically significant differences (*p* < 0.05, ANOVA followed by Tukey’s HSD test)

In the mycelial isolate of *A. crocodilinus*, Ac*ZIP1* gene expression was determined 24 h after exposure to different concentrations of Cd (1 and 2.5 µM) and Zn (100 and 250 µM). The data show that Ac*ZIP1* mRNA levels were significantly downregulated (~ 3-fold) in the presence of non-toxic or sublethal concentrations of Zn (Fig. 7). Although some degree (~ 0.5-fold) of AcZIP1 expression downregulation was detected on sublethal concentrations of Cd, the level was not statistically significant as shown by ANOVA (*p* < 0.05) followed by Tukey’s HSD test.

## Discussion

Analysis of *A. crocodilinus* sporocarp, from which the mycelial strain ACRO2 was isolated, collected at the Morašice locality, showed a high Cd concentration of 149 mg kg⁻¹ dwt (Sácký et al. 2025a), indicating effective Cd accumulation, up to two orders of magnitude above typical mushroom levels (< 1 to 5 mg Cd kg⁻¹ dwt) (Kalač 2019). While *A. crocodilinus* is not classified as a hyperaccumulator, previous studies (Meisch et al. 1977; Cocchi and Vescovi 1997; Melgar et al. 2016) confirm its substantial Cd accumulation potential. Additionally, elevated concentrations of Zn were also previously reported in *A. crocodilinus* (Meisch et al. 1977; Sácký et al. 2025a), prompting us to investigate whether Cd and Zn accumulation and potentially tolerance traits are also present in the mycelial isolate of this species and if they are somehow interlinked.

Mycelia of some species, such as *Amanita muscaria* (Sácký et al. 2022) and *Hebeloma mesophaeum* (Sácký et al. 2019), derived from metal-accumulating fungi collected from clean or polluted sites, when exposed to 5 µM Cd under the same conditions, accumulated 50% less Cd than *A. crocodilinus* mycelium. However, it should be noted that *A. crocodilinus* was significantly less tolerant to Cd, showing more than 60% growth reduction when 5 µM Cd was supplemented in the growth medium, as shown by the metal tolerance assay (Fig. 1). It seems that the mycelium of *A. crocodilinus* from the Morašice region is metal-sensitive, as previous studies in *A. muscaria* and *H. mesophaeum* revealed that metal-tolerant mycelial strains are still viable up to tens of µM Cd or concentrations of Zn in the low millimolar range. Moreover, other studies also showed that metal-tolerant mycelial strains accumulate less metals than sensitive strains (Blaudez et al. 2000a; Colpaert et al. 2005; Ruytinx et al. 2013). Interestingly, exposure to low Zn levels (50 µM) led to a slight, though statistically insignificant, increase in mycelial biomass, suggesting a potential growth-stimulating effect. We have observed this effect previously with mycelium of *H. mesophaeum* isolates exposed to 50 µM of Zn (Sácký et al. 2019). These comparisons indicate that *A. crocodilinus* combines high accumulation capacity with relatively low tolerance, a pattern associated with reduced or disrupted regulation of metal homeostasis and overload of metal-storage within the cells. Fungal tolerance to heavy metals is determined by several mechanisms, including intracellular sequestration of metals in various proteins, such as metallothioneins (MTs) or metal binding peptides, or with glutathione, whose metal complexes can be transported into vacuoles, where they are safely stored (Robinson et al. 2021) or with polyphosphate granules in the case of arbuscular fungi (Ferrol et al. 2016). Other forms of metal storage that could contribute to metal tolerance can be hyphal cell walls (González-Guerrero et al. 2008). Besides that, metal-tolerant fungi can effectively export the accumulated metals from the cells by upregulated efflux systems, such as in the case of *Suillus bovinus* (Ruytinx et al. 2013). While *A. crocodilinus* has been observed to store its extra metals in MTs (Sácký et al. 2025b) or mykophosphatins (Meisch et al. 1983), it likely lacks an effective Cd export mechanism and succumbs to Cd poisoning much quicker than mycelia with either stronger metal-binding and storage mechanisms or effective export mechanisms.

Since the sporocarp and its mycelial isolate clearly displayed a metal accumulation phenotype, and its capacity to store Cd and Zn were described elsewhere (Sácký et al. 2025b), we explored its Zn and Cd uptake modes. Not much is known about the details of Cd uptake in fungi, only that it is active and likely dependent on H^+^ ions, as suggested by experiments on *Paxillus involutus* mycelia (Blaudez et al. 2000b) and that ZIP transporters partly facilitate it. In yeast *Schizosaccharomyces pombe* and *S. cerevisiae*, Cd competes with Zn for “transport tickets” on the Zrt1p transporter (Boch et al. 2008; Rajakumar et al. 2016), and cells become Cd-resistant when the ZRT1 gene is knocked out, because Cd (and Zn) accumulation is decreased. The only *Agaricaceae* transporter where Cd interference with ZIPs was studied is RaZIP1 from *R. bresadolae*. Although this fungus does not accumulate Cd, expressing the Ra*ZIP1* gene in a yeast model leads to increased Cd accumulation (Leonhardt et al. 2018). We thus presumed that in the Cd- and Zn-accumulating *A. crocodilinus*, a ZIP family transporter is also responsible for the accumulation phenotype.

The comparison of the Ac*ZIP1*-cDNA-derived AA sequence revealed that, like its characterized orthologues, RaZIP1 from *R. bresadolae* (Leonhardt et al. 2018) and SlZRT1 from *S. luteus* (Coninx et al. 2017), AcZIP1 contains typical ZIP features described previously, including 8 TMDs, a His-rich loop, in which an intracellular Zn-binding sensor that regulates Zn uptake has been suggested (Pang et al. 2023), and a short C-terminal tail. Both conserved histidyl residues in TMD4 (part of the ZIP signature sequence as defined by Eng et al. 1998) and TMD5 have been shown to be involved in Zn binding and thus essential for ZIP function (Gaither and Eide 2001; Leonhardt et al. 2018; Milon et al. 2006; Rogers et al. 2000). The conservation of key histidyl motifs and regulatory elements in AcZIP1 supports its functional similarity to other ZIP family transporters, making it a strong candidate for mediating the observed Cd accumulation trait.

To confirm the function of AcZIP1 as a Zn and potentially Cd transporter, a functional study was carried out in a yeast model. The yeast studies revealed that AcZIP1 functions as a Zn uptake transporter: Zn-sensitive yeast expressing Ac*ZIP1* showed significantly inhibited growth even on SD medium without Zn addition, while Zn-uptake-deficient yeast became viable with Ac*ZIP1* expression on plain SD medium. No increase in Zn accumulation was observed between 100 and 250 µM Zn^2+^, indicating possible Zn saturation and a loss of AcZIP1 uptake function. Interestingly, a similar effect of slowed down Zn accumulation at higher Zn concentration was observed in the experiment with the mycelial isolate of *A. crocodilinus* (Fig. 2). An in-depth study of *Bordetella bronchiseptica* BbZIP described an internal switch on the loop between TMD3 and TMD4, which regulates Zn transport based on intracellular Zn levels (Pang et al. 2023). This could explain the plateau in Zn accumulation in the yeast model and the mycelium, as AcZIP1 may self-regulate under high Zn. Regarding Cd, constitutive expression of Ac*ZIP1* made Cd-sensitive yeast cells unable to grow at 25 µM Cd²⁺, a level still tolerated by the p416GPD control. This indicates that AcZIP1 is indeed able to transport both Zn and Cd and that AcZIP1 likely contributes to Cd entry into the mycelial cells under natural conditions of low Zn, where its expression remains largely unregulated as shown by the RT-qPCR data (Fig. 7).

Comparative analysis of *A. crocodilinus* AcZIP1 with the previously characterized *Agaricomycetes* ZIPs *R. bresadolae* RaZIP1 (Leonhardt et al. 2018)d *luteus* SlZRT1 (Coninx et al. 2017) indicates that AcZIP1 exhibits an intermediate Zn uptake capacity in yeast at 10 µM Zn (~ 10-fold compared to vector-only control) compared to high-performing RaZIP1 (~ 20-fold compared to vector-only control). Interestingly, SlZRT1 did not increase Zn uptake in yeast nearly to this extent even at 500 µM Zn (~ 2-fold), even though the accumulation was performed for 24 h. Regarding Cd AcZIP1 has a slightly higher Cd-uptake capacity than RaZIP1 (~ 1.44-fold vs. ~1.09-fold at 10 µM Cd compared to vector-only control) while Cd transport of SlZRT1 has not been assessed. This might suggest that AcZIP1 does indeed play a role in the accumulation of Zn and Cd in *A. crocodilinus*. Cd uptake by ZIP proteins has also been reported for fungal RaZIP1 from ectomycorrhizal *R. bresadolae* (Leonhardt et al. 2018), Zrt1p from *S. pombe* (Boch et al. 2008), and Tzn1 and Tzn2 from *Neurospora crassa* (Kiranmayi et al. 2009), where Cd inhibited Zn uptake. We conducted metal accumulation experiments in a yeast model to further support the notion that AcZIP1 mediates Zn and Cd transport to the cytoplasm. Indeed, there was an order of magnitude increase for Zn accumulation compared to the control, but only a twofold increase for Cd accumulation, suggesting a lower capacity of AcZIP1 to transport Cd in the model yeast. Furthermore, Zn ions present in PD medium (2.5 µM) could competitively inhibit Cd binding at the predicted HH-HD site, a motif implicated in Zn binding (Barber-Zucker et al. 2017), thus lowering the total amount of Cd taken up by the yeast. Interestingly, engineering the metal binding site of human hZIP8 (Q180H/E343H) shifted its specificity from multi-metal to Zn-only transport (Jiang et al. 2023). This suggests that AcZIP1 might prefer Zn over Cd and is only partially responsible for the Cd accumulation phenotype in *A. crocodilinus*.

Tagging Ac*ZIP1* with HA increased Zn accumulation (but not Cd accumulation), both in liquid and plate studies, suggesting interference of the tag with transporter function. Although the HA tag appears to alter protein function slightly (Saiz-Baggetto et al. 2017), it remains suitable for the detection of ZIPs, as tagging with larger proteins such as green fluorescent protein (GFP) may be more disruptive (Leonhardt et al. 2018). Fluorescence microscopy showed that AcZIP1 tagged with HA localized to the plasma membrane with some cytoplasmic perturbations. A similar localization was observed with the SlZRT1:GFP fusion protein expressed in yeast (Coninx et al. 2017). The perturbations observed in our study may be caused by the immunofluorescence preparation procedure or could suggest post-translational regulation. Some ZIP proteins undergo endocytic cycling: when Zn levels are high, the protein is internalized and stored in endomembrane vesicles, returning to the membrane under Zn deficiency (Gitan and Eide 2000; Gitan et al. 2003). This may explain the membrane invaginations observed. However, to be certain this is the case, a thorough live microscopy assay must be performed. It should also be noted that the di-leucine endocytosis motif [DE]xxxL[LI] (x for any AA) required for endocytic cycling in human hZIP1 (Huang and Kirschke 2007) is absent from the cytoplasmic loop of AcZIP1. However, in human hZIP2 and hZIP3 the sequence is also absent, yet these undergo endocytic cycling (Bowers and Srai 2018). Thus, it cannot be definitively stated which of these two mechanisms, endocytic cycling or activity blocking, is active in AcZIP1.

ZIP family gene expression typically increases under Zn deficiency and decreases with excess Zn (Coninx et al. 2017; Dainty et al. 2008; Jung 2015; Kiranmayi et al. 2009; Gitan and Eide 2000). Using the mycelial isolate of *A. crocodilinus*, we measured Ac*ZIP1* expression under increasing Zn and Cd concentrations 24 h after exposition. Ac*ZIP1* was strongly downregulated in response to Zn but was only slightly affected by Cd. This aligns with the transcriptional regulation of Sl*ZRT1* in *S. luteus*, where Zn also repressed its expression (Coninx et al. 2017). Direct comparison of a common data point (24-h Zn exposition) could lead to the conclusion that Ac*ZIP1* is more tightly regulated (~ 3.0-fold downregulation already at 100 μm Zn for Ac*ZIP1* compared to only ~ 1.5-fold downregulation at 500 μm Zn for Sl*ZRT1*), however, since the isolates have different metal sensitivity and growth performance and different cultivation techniques were used, it is not possible to draw any definitive conclusions form these observations. The lack of Zn-driven downregulation of Ac*ZIP1* at low Zn and Cd levels suggests that in the case of Cd presence in the environment Cd could enter cells via the transporter, and the fungus would not respond to Cd overload by shutting down Ac*ZIP1* expression. The likelihood of AcZIP1 remaining open for Cd uptake is underscored by the metal accumulation study, as mycelia exposed to 1 µM and 2.5 µM Cd exhibited unimpeded linear accumulation unlike the logarithmic trend observed with Zn. It should be noted that higher levels of Cd and Zn were highly toxic to the mycelium and thus would not reflect the likely situation in unpolluted natural environments, where both Zn and Cd are present in relatively low concentrations (Kubier et al. 2019). Although yeast models have limitations, the observation that Cd was accumulated higher in yeast expressing Ac*ZIP1*, as shown by the yeast plate studies and yeast metal accumulation studies, supports the idea that at least some fraction of Cd enters through AcZIP1, while other transporters likely contribute to the massive Cd uptake in *A. crocodilinus*. As has been postulated before, the CDF transporters, specifically fungal AcCDF2 (Sácký et al. 2025a) and RaCDF2 (Sácký et al. 2016), plant AtZAT1p (Bloss et al. 2002), and human ZnT5B (Valentine et al. 2007) have been shown to uptake Cd. However, the Cd uptake of the fungal transporters, including AcZIP1 has only been proven in the yeast studies and definitive proof coming from observing the protein directly in mycelia, via studies on knock-out mutants or cells expressing mutated proteins, like in the case of human ZnTs (Hoch et al. 2012), or plant ZIPs (Jiang et al. 2023) is still lacking.

## Conclusion

This study shows that the mycelial isolate from a Cd-accumulating *A. crocodilinus* sporocarp is sensitive to Cd and Zn compared to other *Agaricomycetes* isolates, indicating that its accumulation capacity is not accompanied by tolerance. Functional characterization of the AcZIP1 in a yeast model confirmed its role in Zn uptake and suggested a potential role in Cd uptake. The significant downregulation of Ac*ZIP1* in mycelium under Zn excess, but not under Cd exposure, points to limited regulatory control over Cd uptake, which could result in Cd accumulation in the sporocarp through unregulated uptake by AcZIP1. Our research on AcZIP1 bridges the gap between previous *Agaricomycetes* ZIP transporter studies by integrating Cd-related experiments, previously limited to yeast-only systems in the case of *R. bresadolae* RaZIP1, with mycelium-based analyses, which were only performed on Zn-exposed mycelia of *S. luteus*. This comprehensive approach provides a more complete understanding of ZIP transporter function under metal stress in fungi.

## Supplementary Information

Below is the link to the electronic supplementary material.

Supplementary Material 1

Supplementary Material 2

Supplementary Material 3

## Data Availability

Data is provided within the manuscript or supplementary information files.
